# Knowledge of cervical cancer screening among Omani women attending a university teaching hospital: a cross-sectional study

**DOI:** 10.1186/s12905-023-02870-7

**Published:** 2024-01-13

**Authors:** Rahma M Al Kindi, Hana H Al Sumri, Tasneem M Al Muhdhoori, Khoula Al Mamari, Mouza A Al Kalbani, Mohammed H Al-Azri

**Affiliations:** 1 Department of Family Medicine and Public Health, College of Medicine and Health Sciences, Sultan Qaboos University, Muscat, Oman; 2 College of Medicine and Health Sciences, Sultan Qaboos University, Muscat, Oman; 3 Department of Gynecology, Sultan Qaboos Comprehensive Cancer Care and Research Center (SQCCCRC), Muscat, Oman

**Keywords:** Cervical cancer, Papanicolaou test, Cancer screening, Risk factors, Health knowledge, attitudes, Practice, Surveys and questionnaires, Women, Oman

## Abstract

**Objectives:**

To assess knowledge, attitudes, and practices regarding cervical cancer and Pap smear screening among Omani women attending a tertiary clinic in Muscat, Oman, and to establish correlations with selected sociodemographic factors.

**Methods:**

An observational, cross-sectional study was carried out among Omani women aged 18–50 years old attending the outpatient clinic of the Department of Obstetrics and Gynecology, Sultan Qaboos University Hospital, from October 2019 to February 2020. A validated Arabic-language questionnaire was utilized to collect data regarding the participants’ sociodemographic characteristics, their knowledge of cervical cancer and related risk factors, and their knowledge, attitudes, and practices related to cervical cancer screening and Pap smear testing.

**Results:**

Of the 380 respondents, 86 and 55% had previously heard of cervical cancer and Pap smear testing, respectively; however, only 26% were knowledgeable concerning these topics. Knowledge scores were significantly associated with various sociodemographic factors, including marital status and a previous awareness of cervical cancer (odds ratio: > 1, *p* < 0.05). Only 21% had themselves previously undergone Pap smear testing; however, 75% reported being willing to undergo such screening in future.

**Conclusions:**

Knowledge regarding cervical cancer-related risk factors and Pap smear screening was poor among a cohort of Omani women attending a tertiary clinic in Muscat, Oman. This may play a role in the increased frequency of cervical cancer cases observed in Oman over recent years. As such, a well-structured public education program is recommended to raise awareness of this issue.

## Background

Cervical cancer is the fourth most common female cancer globally after breast, colorectal, and lung cancers, and is the second most common female cancer in Low- and Middle-Income Countries (LMICs) after breast cancer [1]. In 2020, it was estimated that approximately 604,127 women were diagnosed with cervical cancer worldwide, with an estimated 341,831 deaths attributable to the disease [2]. The majority (90%) of cervical cancer-related deaths in 2020 occurred in LMICs among women aged 15–49 years, largely due to a lack of access to screening programs, lack of availability of the Human Papillomavirus (HPV) vaccine, poor health infrastructure, and delays in diagnosis [2, 3].

Several risk factors are attributed to the development of cervical cancer, including HPV infection, low socioeconomic status, cigarette smoking, long-term use of oral contraceptives, marriage before the age of 18 years, early sexual intercourse, multiple sexual partners, and multiparity [4, 5]. Nonetheless, HPV infection (primarily genotypes 16 and 18) is considered to be the central etiological risk factor for cervical cancer [6]. According to data sourced from 900 invasive cervical cancer cases originating from 22 countries, 93% of biopsies have HPV DNA [4]. Thus, cervical cancer can be primarily prevented by HPV vaccination and routine Pap smear screening [4, 7].

While many countries have introduced national HPV vaccination programs over the last several decades, such programs remain rare in LMICs [7]. Furthermore, the implementation of Pap smear screening in LMICs is challenging due to poor attendance and lack of awareness of the importance of this type of screening in the prevention and early detection of cervical cancer [8]. Previous studies conducted in LMICs have indicated that women may encounter several barriers to cervical cancer screening, including poor knowledge regarding the importance of the HPV vaccine or Pap smear test, negative attitudes towards cervical cancer-related risk factors (particularly HPV infection), fear of the results, and concern regarding the gender of the doctor performing the screening test [9–11]. In Oman, the HPV vaccine is not yet included in the Expanded National Immunization Program and is therefore not systematically provided by government-funded health institutes under the national Ministry of Health [12, 13].

In Oman, the incidence of cancer has increased over the past 20 years, a finding which may be attributed to population aging, rapid socioeconomic changes, and the increased prevalence of unhealthy lifestyle practices (e.g., tobacco use, physical inactivity, and unhealthy dietary habits), as well as advances in diagnostic and treatment modalities [14]. Among the population of 1.17 million women aged 15 years and older in Oman, there is a notable risk of developing cervical cancer; current estimates suggest that approximately 88 women are diagnosed with cervical cancer each year, resulting in 50 deaths [15]. Moreover, cervical cancer ranks as the fourth most common cancer among women of all ages in Oman and the third most common among women aged 15–44 years [14, 15].

Unfortunately, there are currently no available data on the prevalence of HPV in the general population of Oman. However, in Western Asia, the region to which Oman belongs, it is estimated that about 2.5% of women in the general population are affected by cervical HPV infection at any given time [16]. Furthermore, 72.4% of invasive cervical cancers in this region are attributed to HPV types 16 or 18 [16]. Additionally, a recent study from 2020 indicated that the prevalence of HPV infection remains high in Oman at 17.8% [17].

In Oman, the absence of a well-structured national screening program for cervical cancer and limited availability of Pap smear testing at the primary care level are significant concerns. This lack of accessibility and awareness regarding the availability of the Pap smear test are believed to be the main reasons for the high incidence of cervical cancer in the Middle East region [9, 18]. A previous study indicated that while most Omani women attending a tertiary teaching institute had heard of cervical cancer, they lacked specific knowledge regarding cervical cancer signs and symptoms, risk factors, and Pap smear testing [19]. Therefore, the present study aimed to assess knowledge, attitudes, and practices regarding cervical cancer and Pap smear screening among Omani women attending an outpatient clinic at a tertiary teaching hospital in Muscat, Oman. The study also aimed to establish correlations with various sociodemographic factors. These findings may be useful in informing future health promotion activities that aim to improve cervical cancer awareness in the general public and promote utilization of screening services in Oman.

## Methods

### Study design and setting

An observational, cross-sectional study was carried out from October 2019 to February 2020 at the outpatient clinic of the Department of Obstetrics and Gynecology at the Sultan Qaboos University Hospital (SQUH), Muscat, Oman. As a tertiary hospital, SQUH receives patients from all over the country.

### Study subjects and recruitment strategy

A total of 380 Omani women aged 18–50 years old and attending the clinic for various reasons during the study period were recruited via a systematic random sampling strategy in which every second women registered with the clinic was selected for participation.

### Exclusion and inclusion criteria

The inclusion criteria for the participants included all women of Omani nationality between 15 and 50 years of age attending the outpatient clinic for various reasons. However, those with learning difficulties, those who did not speak Arabic or English, those with emergency conditions or who were very sick, and those with no time to participate in the survey were excluded.

### Sample size

The necessary sample size was calculated to be 374 women, based on an anticipated level of knowledge regarding cervical cancer and its screening (50%), with a 5% margin of error, 95% confidence level, 5% alpha error, and a design effect of 2.

### Survey instrument

A validated, pre-tested, Arabic-language questionnaire was used for data collection purposes. The questionnaire had been previously used in similar studies performed in Oman [19–22]. This four-part questionnaire was self-administered and took approximately 15–20 minutes to complete. The first section covered the participants’ sociodemographic characteristics, including their age, education level, employment status, marital status, number of marriages, number of previous pregnancies, their husbands’ education level, and monthly family income. The second section assessed the presence of known risk factors for cervical cancer, such as smoking status, personal history of cervical cancer, and family history of cervical cancer.

The third part of the questionnaire assessed the participants’ knowledge regarding cervical cancer, related risk factors, and appropriate screening. This section covered whether the participants had ever heard of cervical cancer and whether they believed that cervical cancer can be prevented, has a latent and asymptomatic period, can be detected in its early stages, is curable when detected early, is a genetic disease, is more likely if a family member has it, can be prevented by maintaining healthy sexual hygiene, if postmenopausal women and HPV-positive women are at risk of getting cervical cancer, whether cytological examination is the main screening method in the early stages of the disease, and whether the disease is caused by a specific HPV genotype. In addition, this section explored knowledge of cervical cancer-related risk factors, including HPV infection, early sexual activity, multiple sexual partners, multiparity, and smoking status. All questions in the second and third sections of the questionnaire were designed to elicit yes/no responses.

The last part of the questionnaire evaluated the participants’ knowledge, attitudes, and practices related to cervical cancer screening and Pap smear testing. Participants were asked if they had ever heard of Pap smear screening, if they had previously undergone Pap smear testing, and whether they would be willing to undergo such testing. In turn, those who had never undertaken Pap smear testing were asked about their reasons for not doing so and their willingness to undertake such screening in the future. Awareness of the actual screening procedure was assessed, with participants being asked about the aim, usefulness, and importance of screening, the appropriate time for testing, the site of the test, whether one needed to be symptomatic to get the test, and when to stop the test. Most of the questions in this section also required yes/no responses; however, there were exceptions for certain questions, such as reasons for not taking action, screening aims, appropriate time for testing, site of testing, and when to stop the test. For these questions, respondents were provided with multiple-choice options to choose from.

### Scoring

All knowledge-related items in the questionnaire were compiled, and a scoring system was created. Each correct response received a score of 1, resulting in a total score range of 0–30. Patients were then divided into two categories based on their total scores: not knowledgeable (scores of ≤15) and knowledgeable (scores of 16–30).

### Ethics

Ethical approval for this study was obtained from the Medical Research and Ethics Committee of the College of Medicine and Health Sciences, Sultan Qaboos University (#SQU-EC/214/19, #MREC2013). All participants were briefed regarding the objectives of the study and were informed that their participation was voluntary in nature and that they had the right to withdraw at any time. Written informed consent was received from all of the women prior to their participation in the study. The participants’ anonymity and confidentiality were ensured at all times, with each participant assigned a unique identification number for the purposes of data analysis.

### Statistical analysis

The data analysis was carried out using the Statistical Package for the Social Sciences (SPSS), version 23 (IBM Corp., Armonk, NY). Descriptive statistics were used to report the sample’s characteristics. For categorical variables, frequencies and percentages were reported, whereas means and standard deviations were used to present continuous variables. Crude and adjusted Odds Ratios (ORs) and corresponding 95% Confidence Intervals (CIs) were used to test correlations, and a *p* value of ≤0.05 was considered statistically significant.

## Results

### Sociodemographic characteristics

The mean age was 32.1 ± 7.6 years (range: 18–50 years), with a similar proportion of participants aged 18–30 years (*n* = 182; 48%) and 31–50 years (*n* = 198; 52%). More than half of the participants had an undergraduate-level education or higher (*n* = 247; 65%). Most were unemployed (*n* = 222, 58%) and had been married once (*n* = 290; 76%), with few having been married more than once (*n* = 7; 2%). Of the participants who had been married at least once, the majority of their husbands had secondary school diplomas (*n* = 149; 51%) or undergraduate degrees (*n* = 141; 49%). In terms of family income, 209 (55%) reported earning < 1000 Omani Riyals (OMR) per month (equivalent to <$2598 USD). The vast majority were non-smokers (*n* = 377; 99%). Only two participants (1%) had a personal history of cervical cancer, while only one (< 1%) was aware of a family history of cervical cancer (Table 1).

**Table 1:** Selected sociodemographic characteristics of Omani women attending a tertiary clinic in Muscat, Oman (*N* = 380)

Characteristic	n (%)
**Age (years)**	
18–30	182 (47.9)
31–50	198 (52.1)
**Education level**	
Secondary	133 (35.0)
Undergraduate or higher	247 (65.0)
**Employment status**	
Unemployed	222 (58.4)
Employed	158 (41.6)
**Marital status**	
Never married	90 (23.7)
Married once	283 (74.5)
Married more than once	7 (1.8)
**Monthly income (OMR)**	
< 1000^a^	209 (55.0)
≥ 1000	171 (45.0)
**Number of previous pregnancies**	
0	112 (29.7)
1–2	91 (23.9)
≥ 3	176 (46.3)
**Previously heard of cervical cancer**	
Yes	325 (85.5)
No	55 (14.5)

*OMR* Omani Riyals. ^a^Equivalent to <$2598 USD

### Knowledge of cervical cancer and pap smear testing

In terms of cervical cancer-related knowledge, most participants (*n* = 325; 86%) had previously heard of cervical cancer. Over half (*n* = 224; 59%) believed that cervical cancer can be prevented and 251 (66%) thought it a genetic disease. The most frequently identified cervical cancer-related risk factors included multiple sexual partners (*n* = 162; 43%), smoking (*n* = 145; 38%), and HPV infection (*n* = 91; 24%). Early sexual activity (*n* = 41; 11%) and having three or more children (*n* = 30; 8%) were the least frequently reported risk factors.

With regards to knowledge, attitudes, and practices related to Pap smear testing, 209 women (55%) had previously heard of this screening method, although the majority (*n* = 302; 80%) admitted that they did not undertake such testing on a regular basis. Only 78 women (21%) had themselves previously undergone Pap smear testing, although 283 (75%) reported a willingness to undergo such testing in the future. Various reasons were reported for not having previously undertaken Pap smear testing, including concern regarding being examined by a male doctor (*n* = 287; 76%), feeling embarrassed (*n* = 183; 48%), fear of the test results (*n* = 172; 45%), fear of pain (*n* = 139; 37%), being healthy (*n* = 136; 36%), being busy (*n* = 115; 30%), fear of the test itself (*n* = 98; 26%), the unavailability of nearby health services (*n* = 89; 23%), being discouraged by their partner (*n* = 88; 23%), privacy concerns (*n* = 87; 23%), fear of bleeding (*n* = 69; 18%), being unaware of where to get the test (*n* = 50; 13%), having no time (*n* = 49; 13%), the expense of being tested (*n* = 45; 12%), religious reasons (*n* = 44; 12%), and being too old to be tested (*n* = 34; 9%).

### Associations with sociodemographic characteristics

Overall, the vast majority of participants (*n* = 281; 74%) were not knowledgeable with regards to cervical cancer and Pap smear testing, receiving total scores of < 16 for all knowledge-related items in the questionnaire. Only 99 (26%) women were considered knowledgeable on these topics, with total scores of ≥16. Knowledge scores were significantly associated with several sociodemographic factors, including marital status (OR 2.84, 95% CI 1.42–5.67) and a previous awareness of cervical cancer (OR 4.82, 95% CI 1.68–13.83), with married women, those who had had one or two pregnancies, and those who had previously heard of cervical cancer being more knowledgeable compared to their respective counterparts. No significant associations were observed between knowledge scores and other sociodemographic characteristics of the participants, including age, educational and employment status, monthly income level, and number of previous pregnancies (*p* > 0.05) (Table 2).

**Table 2:** Associations between sociodemographic characteristics and knowledge, attitudes, and practices related to cervical cancer and Pap smear testing among Omani women attending a tertiary clinic in Muscat, Oman (*N* = 380)

Characteristics	Knowledge of cervical cancer^a^		Adjusted Odds Ratio (95% CI)
	Good	Poor	
**Age (years)**			0.71 (0.43–1.18)^b^
18–30	44 (44.4)	138 (49.1)	
31–50	55 (55.6)	143 (50.9)	
**Education level**			1.22 (0.74–2.02)^d^
Secondary	30 (31.3)	103 (36.3)	
Undergraduate or higher	66 (68.8)	181 (63.7)	
**Employment status**			0.91 (0.56–1.48)^d^
Unemployed	56 (58.3)	166 (58.5)	
Employed	40 (41.7)	118 (41.5)	
**Marital status**			2.84 (1.42–5.67)^c^
Never married	13 (13.5)	77 (27.1)	
Married once or more	83 (86.5)	207 (72.9)	
**Monthly income (OMR)**			0.90 (0.56–1.45)^d^
< 1000	56 (58.3)	153 (53.9	
≥ 1000	40 (41.7)	131 (46.1)	
**Number of prior pregnancies**			0.95 (0.38–2.42)^d^
0	20 (20.8)	93 (32.7)	
≥ 1	76 (79.2)	191 (67.3)	
**Previously heard of cervical cancer**			4.82 (1.68–13.83)^d^
No	3 (3.0)	52 (18.5)	
Yes	96 (97.0)	229 (81.5)	

^a^Assessed using a validated, pre-tested, Arabic-language questionnaire [17–20]. Total scores of ≤15 and 16–30 were considered to indicate poor and good knowledge of cervical cancer, respectively. ^b^adjusted for marital status only; ^c^adjusted for age only; ^d^adjusted for age and marital status

Furthermore, significant associations were noted between Pap smear practices and several factors, including age (OR 2.13, 95% CI 1.18–3.84), marital status (OR 21.76,, 95% CI 2.91–162.63), and a previous awareness of cervical cancer (OR 2.44, 95% CI 0.90–6.54). Specifically, older women, those who were married, and those who had previously heard of cervical cancer more frequently reported having previously undertaken Pap smear testing compared to those who were younger, those unmarried, and those who had not heard of cervical cancer before (Table 3). Moreover, married women (OR 4.56, 95% CI 2.52–8.25) and those who had heard of cervical cancer before (OR 2.42, 95% CI 1.29–4.52) more frequently reported a willingness to undertake such testing in the future (Table 4).

**Table 3:** Associations between sociodemographic characteristics and practice related to cervical cancer and Pap smear testing among Omani women attending a tertiary clinic in Muscat, Oman (*N* = 380)

Characteristic	Previously undergone Pap smear testing		Adjusted Odds Ratio (95% CI)
	Yes	No	
**Age (years)**			**2.13 (1.18–3.84)**^**a**^
18–30	19 (24.4)	163 (54.0)	
31–50	59 (75.6)	139 (46.0)	
**Education level**			1.03 (0.59–1.77)^c^
Secondary	61 (29.2)	72 (42.1)	
Undergraduate or higher	148 (70.8)	99 (57.9)	
**Employment status**			1.16 (0.69–1.96)^c^
Unemployed	117 (56.0)	105 (61.4)	
Employed	92 (44.0)	66 (38.6)	
**Marital status**			**21.76 (2.91–162.63)**^**b**^
Never married	27 (12.9)	63 (36.8)	
Married once or more	182 (87.1)	108 (63.2)	
**Monthly income (OMR)**			1.09 (0.64–1.85)^c^
< 1000	113 (54.1)	96 (56.1)	
≥ 1000	96 (45.9)	75 (43.9)	
**Number of prior pregnancies**			0.58 (0.23–1.46)^c^
0	9 (11.5)	104 (34.4)	
≥ 1	69 (88.5)	198 (65.6)	
**Previously heard of cervical cancer**			**2.44 (0.90–6.54)**^**c**^
Yes	73 (93.6)	50 (16.6)	
No	5 (6.4)	252 (83.4)	

^a^Adjusted for marital status only; ^b^Adjusted for age only; ^c^Adjusted for age and marital status

**Table 4:** Associations between sociodemographic characteristics and attitude related to cervical cancer and Pap smear testing among Omani women attending a tertiary clinic in Muscat, Oman (*N* = 380)

Characteristic	Willingness to undertake Pap smear testing in future		Adjusted Odds Ratio (95% CI)
	Yes	No	
**Age (years)**			0.89 (0.51–1.56)^a^
18–30	16 (26.2)	166 (52.0)	
31–50	45 (73.8)	153 (48.0)	
**Education level**			1.49 (0.90–2.47)^c^
Secondary	93 (32.9)	40 (41.2)	
Undergraduate or higher	190 (67.1)	57 (58.8)	
**Employment status**			1.07 (0.65–1.78)^c^
Unemployed	160 (56.5)	62 (63.9)	
Employed	123 (43.5)	35 (36.1)	
**Marital status**			**4.56 (2.52–8.25)**^**b**^
Never married	46 (16.3)	44 (45.4)	
Married once or more	237 (83.7)	53 (54.6)	
**Monthly income (OMR)**			1.20 (0.74–1.97)^c^
< 1000	154 (54.4)	55 (56.7)	
≥ 1000	129 (45.6)	42 (43.3)	
**Number of prior pregnancies**			0.19 (0.52–1.62)^c^
0	7 (11.5)	106 (33.2)	
≥ 1	54 (88.5)	213 (66.8)	
**Previously heard of cervical cancer**			**2.42 (1.29–4.52)**^**c**^
Yes	57 (93.4)	268 (84.0)	
No	4 (6.6)	51 (16.0)	

^a^Adjusted for marital status only; ^b^adjusted for age only; ^c^Adjusted for age and marital status

## Discussion

Although cervical cancer is the one of the most common cancers among Omani women, there is as yet no established cervical cancer screening program in the country [12–15]. Moreover, Pap smear testing is often only provided to married women and is unavailable at the primary healthcare level, performed solely in secondary and tertiary care facilities for diagnostic purposes. Combined with the lack of a national HPV vaccination program, such concerns are critical because the onus for cervical cancer screening and Pap smear testing therefore rests on the individual patient. As such, public knowledge and awareness of the importance of such screening and cervical cancer risk factors is crucial for early identification and prevention purposes. Thus, the present study was conducted in order to assess knowledge, attitudes, and practices regarding cervical cancer and Pap smear screening and to establish correlations with various sociodemographic factors among a cohort of Omani women attending an outpatient clinic at a tertiary teaching hospital in Muscat, Oman. The findings indicated that cervical cancer knowledge was limited, with 86% having heard of it, but only 59% believing in its preventability. Regarding Pap smear testing, 55% were aware, but 80% did not undergo such testing regularly. Sociodemographic factors such as marital status and previous awareness of cervical cancer were associated with higher knowledge scores and increased likelihood of participating in Pap smear practices.

In our study, although the vast majority of participants (86%) had previously heard of cervical cancer, only 55% had heard of Pap smear testing. A prior survey conducted in Oman similarly reported that 80% of participants had heard of cervical cancer [19]. This level of public awareness is high, possibly because such studies were conducted among patients, students, and employees at a tertiary care institute that routinely provides this service. According to Shrestha et al., only 18% of women who visited a tertiary care facility in Nepal knew of Pap smear testing, despite 66% having previously heard of cervical cancer [23]. In contrast, much higher rates of awareness (85 and 76%, respectively) were recorded among women accessing primary healthcare facilities in Qatar [24]. In Kuwait, 77% of married women visiting polyclinics had heard of Pap smear testing; similarly, 74% of Vietnamese American women surveyed in the USA had heard of the Pap smear test, although 90% were aware of cervical cancer [25, 26]. Differences in knowledge across different countries or regions may be due to cultural or healthcare system factors. For example, although Qatar is also a Middle Eastern country with a similar sociocultural milieu to Oman, the researchers noted that Pap smear testing is available at most primary health centers due to the country’s well-woman clinic program [24].

In the current study, various sociodemographic factors were found to have a significant impact on knowledge of cervical cancer and Pap smear testing, including marital status and a previous awareness of cervical cancer. Similar findings have been reported in studies conducted in Qatar, Kuwait, and the USA [24–26]. In particular, married women were significantly more knowledgeable compared to their counterparts, as well as those who had previously heard of cervical cancer. Marital status likely influences cervical cancer-related knowledge in Oman due to family planning practices among married women, which would involve regular consultations with an obstetrician or gynecologist. These healthcare professionals often provide guidance on fertility, prenatal care, and childbirth preparation, possibly encompassing discussions about women’s general reproductive health, including cervical cancer. Indeed, this would tie in with the finding that knowledge scores were significantly associated with number of previous pregnancies. Studies from Kazakhstan have similarly shown that number of births is associated with increased awareness of HPV and cervical cancer screening practices [27, 28]. Indeed, the impact of marital status on cervical cancer-related knowledge may be more pronounced in conservative, religious countries like Oman where premarital sexual relations are strictly prohibited. Alternatively, as previously described, Pap smear testing is often offered only to married women in Oman; this might also explain why this group were more likely to demonstrate knowledge of cervical cancer and Pap smear testing. However, previous studies from Oman have also reported associations with other factors, such as education level, employment status, and income [19–22]. These variations in findings could be due to differences in the research population and study design, as well as in the availability and accessibility of cervical cancer screening services in different healthcare settings and regions.

In terms of knowledge regarding cervical cancer, our study reported results in line with previous studies performed in Qatar and Kuwait [21, 22]. However, given that the majority of the participants (74%) had inadequate knowledge regarding cervical cancer and Pap smear testing, regardless of education level, this may indicate that the Omani public’s overall exposure to cervical cancer-related education is lacking. Unfortunately, only 24% of participants in the current study were aware of HPV infection as a risk factor for cervical cancer. This is concerning as previous research has identified a link between awareness of HPV as a major cause of cervical cancer and participation in Pap smear practices [27, 28]. Only 21% of the women in our study revealed that they had previously undergone Pap smear testing themselves. Despite this, the majority (75%) reported that they would be willing to undergo such testing in the future,

One of the limitations of this study includes the length of the questionnaire and the fact that the questionnaires were completed by participants while in the clinic’s waiting area, which can often be noisy and crowded. Moreover, some of the questions sought information on the participants’ past experiences and were related to potentially sensitive topics which could have resulted in recall or response bias. Secondly, the study was conducted in a single institution located in the capital city of Muscat; accordingly, the findings cannot be generalized to other institutions or regions of Oman, particularly more remote regions. Nonetheless, a strength of the study was that the participants involved in the study originated from all over the country as SQUH is one of the few tertiary-level institutions in Oman.

Overall, the present study identified inadequate awareness on matters related to cervical cancer, its risk factors, and screening methods among a sample of Omani women attending a tertiary outpatient clinic. Accordingly, more efforts need to be made to increase general awareness of cervical cancer and its screening methods and risk factors through various channels, including school-level interventions and health promotion activities. In particular, healthcare professionals should be encouraged to deliver cervical cancer-related information to female patients of appropriate age groups as they would be able to immediately and accurately respond to any inquiries and correct any misconceptions. Commonly reported barriers to taking part in cervical cancer screening in Oman include fear of being examined by a male doctor, being embarrassed, fear of the results, fear of pain, being healthy, being busy, and fear of the procedure itself [19–22]. It is likely that these women would demonstrate more positive attitudes concerning Pap smear testing if they were to be reassured and given more detailed information concerning the test procedure itself. Much of this responsibility is likely to fall on the attending physician who should provide detailed information to female patients regarding cervical cancer screening, including its indications, ideal frequency, and possible complications. Moreover, healthcare professionals—whether at the primary, secondary, or tertiary care level—should seek to maintain appropriate channels of communication throughout the entire consultation and follow-up process in order to build up a trusting relationship with their patients, particularly given that such topics may be considered sensitive or embarrassing, especially in very conservative, religious societies.

## Conclusions

The burden imposed by cervical cancer on health systems remains considerable, especially in LMICs such as Oman which lack adequate HPV vaccination and cervical cancer screening coverage. As such, public awareness of these issues is paramount to ensure early identification and prevention. Overall, the present study identified suboptimal awareness on matters related to cervical cancer, its risk factors, and screening methods among a cohort of Omani women attending a tertiary outpatient clinic. Despite this, most participants were generally accepting of the idea of undergoing Pap smear testing in future. Thus, it is strongly recommended that the Omani Ministry of Health consider implementing a national screening program in order to lower the incidence of cervical cancer. Moreover, awareness and education campaigns are also urgently needed to effectively inform the public on the importance of and indications for cervical cancer screening. Such campaigns might be implemented utilizing a multimedia approach; in addition, collaboration with other government sectors, such the Ministry of Education, would be helpful in order to reach adolescents and younger women and to incorporate knowledge about cervical cancer warning signs and risk factors into national school curricula. To support the execution of such initiatives, further research is recommended, particularly studies exploring ways to reduce barriers to Pap smear screening and the impact of religious and cultural factors on cervical cancer awareness and screening practices. In addition, comprehensive research is needed to determine the prevalence of HPV infection in the general population of Oman, with a focus on identifying specific genotypes. This could inform decisions related to the inclusion of the HPV vaccine in national immunization strategies.

## Data Availability

The raw datasets used and/or analyzed during this study are available from the corresponding author upon reasonable request.

## Abbreviations

HPV: Human Papillomavirus
LMICs: Low- and Middle-Income Countries
OMR: Omani Riyals
Pap: Papanicolaou
SQUH: Sultan Qaboos University Hospital
SPSS: Statistical Package for the Social Sciences
OR: Odds Ratio
CI: Confidence Interval
USA: United States of America
